# Ride comfort and segmental vibration transmissibility analysis of an automobile passenger model under whole body vibration

**DOI:** 10.1038/s41598-023-38592-x

**Published:** 2023-07-18

**Authors:** Veeresalingam Guruguntla, Mohit Lal, G. S. Pradeep Ghantasala, P. Vidyullatha, Malak S. Alqahtani, Najah Alsubaie, Mohamed Abbas, Ben Othman Soufiene

**Affiliations:** 1 Department of Industrial Design, National Institute of Technology Rourkela, Rourkela, Odisha India; 2 Chitkara University Institute of Engineerin and Technology, Chitkara University, Rajpura, Punjab India; 3 Department of CSE, Koneru Lakshmaiah Education Foundation, Vaddeswaram Guntur, AP India; 4 Computer Engineering Department, College of Computer Science, King Khalid University, Abha, 61421 Saudi Arabia; 5 Department of Computer Sciences, College of Computer and Information Sciences, Princess Nourah Bint Abdulrahman University, P.O. Box 84428, Riyadh, 11671 Saudi Arabia; 6 Electrical Engineering Department, College of Engineering, King Khalid University, Abha, 61421 Saudi Arabia; 7 Prince Laboratory Research, ISITcom, University of Sousse, 4023 Hammam Sousse, Tunisia

**Keywords:** Biotechnology, Computational biology and bioinformatics, Engineering

## Abstract

The examination of seated occupants’ ride comfort under whole-body vibration is a complex topic that involves multiple factors. Whole-body vibration refers to the mechanical vibration that is transmitted to the entire body through a supporting surface, such as a vehicle seat, when traveling on rough or uneven surfaces. There are several methods to assess ride comfort under whole-body vibration, such as subjective assessments, objective measurements, and mathematical models. Subjective assessments involve asking participants to rate their perceived level of discomfort or satisfaction during the vibration exposure, typically using a numerical scale or questionnaire. Objective measurements include accelerometers or vibration meters that record the actual physical vibrations transmitted to the body during the exposure. Mathematical models use various physiological and biomechanical parameters to predict the level of discomfort based on the vibration data. The examination of seated occupants ride comfort under whole-body vibration has been of great interest for many years. In this paper, a multi-body biomechanical model of a seated occupant with a backrest is proposed to perform ride comfort analysis. The novelty of the present model is that it represents complete passenger by including thighs, legs, and foot which were neglected in the past research. A multi-objective firefly algorithm is developed to evaluate the biomechanical parameters (mass, stiffness and damping) of the proposed model. Based on the optimized parameters, segmental transmissibilities are calculated and compared with experimental readings. The proposed model is then combined with a 7-*dofs* commercial car model to perform a ride comfort study. The ISO 2631-1:1997 ride comfort standards are used to compare the simulated segmental accelerations. Additionally, the influence of biomechanical parameters on most critical organs is analyzed to improve ride comfort. The outcomes of the analysis reveal that seated occupants perceive maximum vibration in the 3–6 Hz frequency range. To improve seated occupants' ride comfort, automotive designers must concentrate on the pelvis region. The adopted methodology and outcomes are helpful to evaluate protective measures in automobile industries. Furthermore, these procedures may be used to reduce the musculoskeletal disorders in seated occupants.

## Importance of the study

**Figure 1:**
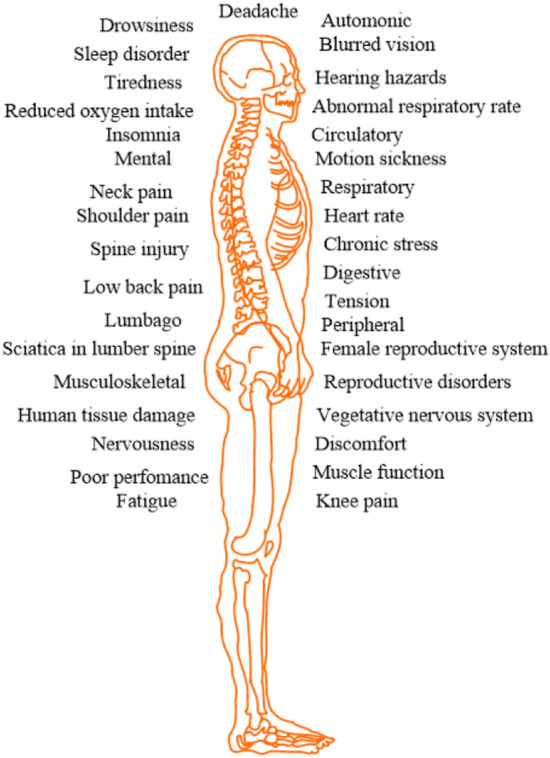
Disorders and problems arise due to WBV^4^.

Occupants (drivers or passengers) using various transportation means like motorbikes, cars, subways, aircraft, ferries, levitated vehicles, and high-speed bullet trains expose to unaccountable vibrations during their daily life transportation. These vibrations may be passed to the complete human body or to specific segments/parts of the body via a supporting structure such as a car seat, tractor, or ship^1^. This transmitted vibration is called whole-body vibration (WBV). Drivers occupy awkward postures to perform various control operations that cause *WBV* and lead to low-back pain and musculoskeletal disorders (MSDs). The long-term vibration could affect renal function^2^. The study^3^ discussed the reasons for modeling the human and the necessity of dynamic response prediction in *WBV* analysis. The authors in^4^ estimated the segmental vibration transmissibility under various input excitation conditions. In moving vehicles, tragic incidents happen every year and in industrial workers (see Fig. 1).

A sustainable review of mathematical models for human modeling is presented in^5^. Among all 4-*dofs* models, the model developed by Wan and Chimes is suggested to precisely predict the magnitude of biodynamic responses^6^. The authors in^7^ optimized the parameters of the model proposed in^8^ by changing the number of springs and dampers, keeping the number of masses constant. Another work in^9^ improved the efficiency of Wan model^6^ with those parameters the acceleration received from seat to various segments is analyzed.

The authors in^10^ proposed 6-*dofs* multi body (MB) model with an inclined backrest to mimic seated occupant. Marzbanrad et al.^11^ developed a 6-*dofs* model of a seated occupant with a vertical backrest. By incorporating both vertical and fore-aft vibrations, a 7-*dofs* biomechanical model is developed by Gan et al.^12^. Another study in^13^ developed a 7-*dofs* biomechanical model by assuming each human segment is connected with a rotational spring and damper. The developed model was suitable for estimating both apparent mass (AM) and seat-to-head-transmissibility (STHT). Sensitivity analysis reveals that lower body properties affect the peak value of apparent mass.

The authors in^14^ implemented 9-*dofs* MB model by adding backrest and calculated STHT and back to head transmissibility and validated with experiments. Similarly, by partitioning the human body into three parts, Cho and Yoon^15^ constructed a 9-*dofs* MB model of a seated human with backrest support. This model was stretched to 14-*dofs* by the authors in^16^ improved the model efficiency. Recently, the authors in^17^^,^^18^ developed 10-*dofs* biomechanical model to mimic real human structure. With modal and sensitive analysis, they observe the magnitude of biodynamic responses that get affected more by pelvis and least by head parameters.

The need to build a more efficient coupled human-vehicle model was piqued by the requirement for ride comfort. With this objective, Reddy et al.^19^ created a 15-dofs sitting human model and combined it with a 7-dofs entire car model to determine low-frequency ride comfort values. Furthermore, Zoccali et al.^20^ and Castellanos and Fruett^21^ performed ride comfort analysis on Italians. Recently^22^ carried out ride comfort analysis on tractor drivers while performing rotary soil tillage operations. In another study^23^ used the deep learning method to investigate the relation between car speed and road conditions on ride comfort.

According to the literature, the structure of the multi body model plays an important role in analyzing and simulating biodynamic responses. In the past, a number of MB models have developed to represent seated human occupants but none of these models considered thighs, legs and feet, in this way the present work is novel. Since the dynamic behaviour of the seated human is characterized equally by seat-to-head transmissibility (STHT) and apparent mass (AM). Hence, to make the analysis more realistic the magnitude and phase of STHT and AM are considered in the development of an objective function. Later, sensitivity analysis was performed to estimate the transmissibility ratio (TR) among seat to various human segments (head, thorax, abdomen and pelvis). Additionally, the proposed model coupled with a full car model to analyze the ride dynamic behaviour of human beings under low-frequency vibration (< 20 Hz) based on ISO 2631-1:1997 charts^24^. The obtained data will help the designers to improvise the ride comfort of human beings.

## Model characteristics

The following assumptions/limitations are considered in the present study:

- Only the effect of vertical vibration on different biodynamic responses as well as different segments are considered because vertical direction is the most pronounced and dominant direction of vibration due to road profiles (humps, uneven surface, crooked roads etc.). It may also be acknowledged that all the suspension systems provided in automobiles are to compensate this vertical vibration.
- In addition, most of the standards such as International Standard Organization (ISO-2631), British Standards (BS-6841), and European standards EN-12,299 acknowledged that the human body is more sensitive and susceptible to the vertical vibration in comparison with fore-and-aft direction.
- Also simultaneous estimation in multiple directions has practical limitation of number of sensors and measuring locations. As a result, it leads to more complex instrumentation, data acquisition system and data analysis techniques.

**Figure 2:**
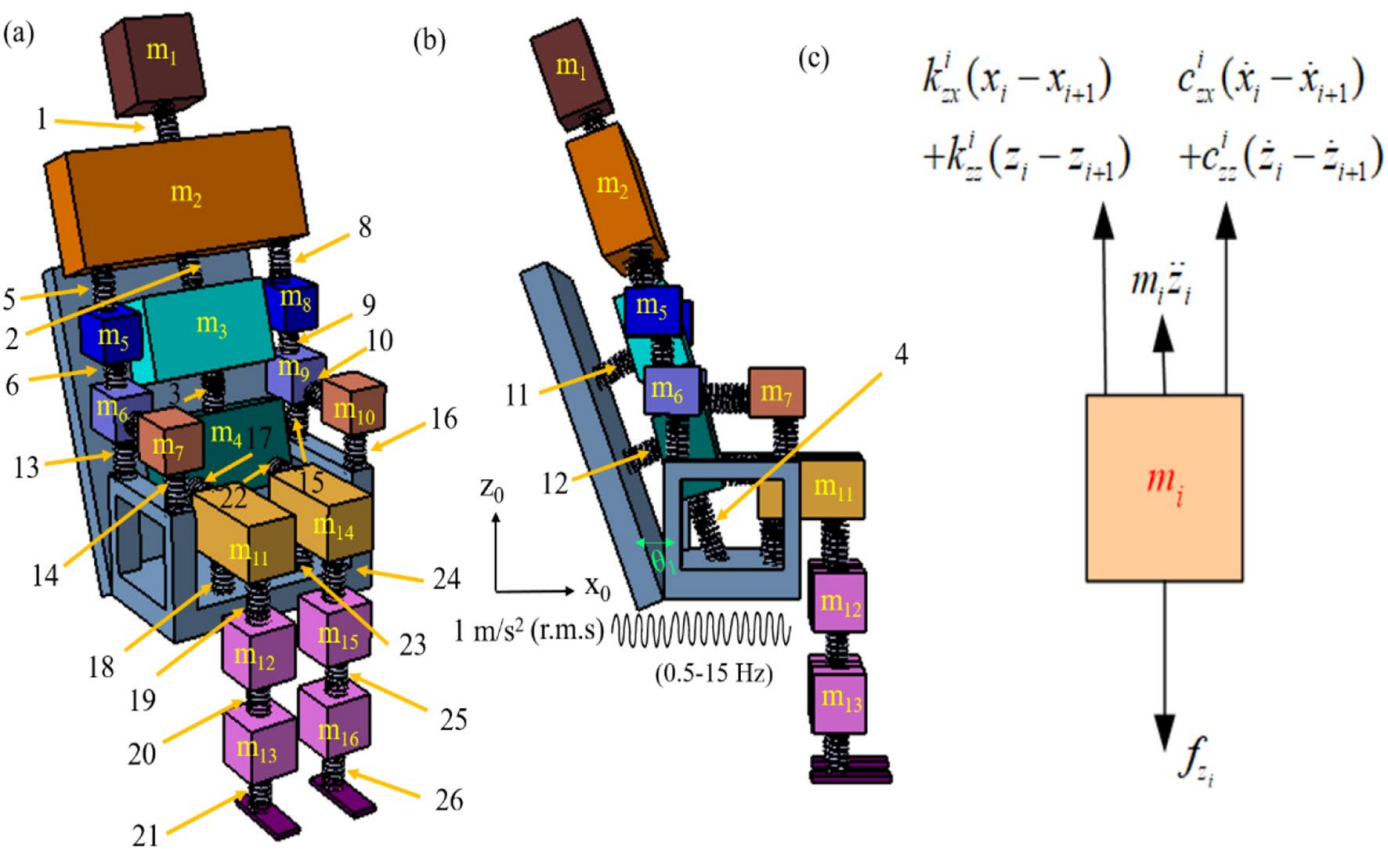
The schematic representation of passenger model in (*z*–*x*) plane (**a**) isometric view (**b**) left side view (**c**) free body diagram of *i*th segment.

With these assumptions a schematic of 32-*dofs* biomechanical model of a seated human is presented in Fig. 2. The human body is imaginarily segmented, and each segment is connected with an adjacent segment via stiffness and damping characteristics that are both direct and cross-coupled. Since the human body is symmetric about the sagittal plane. Hence, in the modeling the biomechanical properties (mass, stiffness and damping) are considered symmetric about the sagittal plane. In addition, the cross-coupled parameters are considered symmetric. The model consists of different segments (*i* = 1–16) that represents anatomy of head (*m*_*1*_), thorax (*m*_*2*_), abdomen (*m*_*3*_), pelvis (*m*_*4*_), upper arms (*m*_*5*_ and *m*_*8*_), forearms (*m*_*6*_ and *m*_*9*_), hands (*m*_*7*_ and *m*_*10*_), thighs (*m*_*11*_ and *m*_*14*_), legs (*m*_*12*_ and *m*_*15*_), feet (*m*_*13*_ and *m*_*16*_) (refer Fig. 2). To avoid complexity in representation only springs are shown in Fig. 2, whereas dampers can also be shown alongside springs. Direct and cross-coupled stiffness (*K*_*ij*_) and damping (*C*_*ij*_) qualities exist for each spring and damper (*i* = *x*, *j* = *z*; where ‘*x’* and ‘*z’* indicate fore-and-aft and vertical direction, respectively).

### Governing equations

By applying Newton's second law to each section, the system's governing equations are formulated (refer to Fig. 2c). For brevity and completeness, the governing equation for ‘*i*th’ segment connected to ‘*i* + 1th’ segment may present as:

1 $$m_{i} \ddot{x}_{i} + c_{xx}^{i} (\dot{x}_{i} - \dot{x}_{i + 1} ) + c_{xz}^{i} (\dot{z}_{i} - \dot{z}_{i + 1} ) + k_{xx}^{i} (x_{i} - x_{i + 1} ) + k_{xz}^{i} (z_{i} - z_{i + 1} ) = f_{{x_{i} }}$$

2 $$m_{i} \ddot{z}_{i} + c_{zx}^{i} (\dot{x}_{i} - \dot{x}_{i + 1} ) + c_{zz}^{i} (\dot{z}_{i} - \dot{z}_{i + 1} ) + k_{zx}^{i} (x_{i} - x_{i + 1} ) + k_{zz}^{i} (z_{i} - z_{i + 1} ) = f_{{z_{i} }}$$

The above Eqs. (1, 2), may present in matrix form as,

3 $$[M_{i} ]\left\{ {\ddot{\chi }_{i} } \right\} + [C_{i} ]\left\{ {\dot{\chi }_{i} - \dot{\chi }_{i + 1} } \right\} + [K_{i} ]\left\{ {\chi_{i} - \chi_{i + 1} } \right\} = \{ f_{i} \}$$

where

$$[M_{i} ] = \left[ {\begin{array}{*{20}c} {m_{i} } & 0 \\ 0 & {m_{i} } \\ \end{array} } \right],[C_{i} ] = \left[ {\begin{array}{*{20}c} {c_{xx}^{i} } & {c_{xz}^{i} } \\ {c_{zx}^{i} } & {c_{zz}^{i} } \\ \end{array} } \right],[K_{i} ] = \left[ {\begin{array}{*{20}c} {k_{xx}^{i} } & {k_{xz}^{i} } \\ {k_{zx}^{i} } & {k_{zz}^{i} } \\ \end{array} } \right],\{ f_{i} \} = \left\{ {\begin{array}{*{20}c} {f_{{x_{i} }} } \\ {f_{{z_{i} }} } \\ \end{array} } \right\},\{ \chi_{i} \} = \left\{ {\begin{array}{*{20}c} {x_{i} } \\ {z_{i} } \\ \end{array} } \right\}$$

After developing equations of motion (EOMs) for each segment, the global EOM may express as,

4 $$[M]_{16 \times 16} \left\{ {\ddot{\chi }} \right\}_{16 \times 1} + [C]\left\{ {\dot{\chi }} \right\}_{16 \times 1} + [K]\left\{ \chi \right\}_{16 \times 1} = \{ f\}_{16 \times 1}$$

Now by substituting *χ* = **χ***e*^*jωt*^ and *f* = *Fe*^*jωt*^ in Eq. (4), the time series equation may convert into frequency series as,

5 $$( - \omega^{2} M + j\omega C + K)_{16 \times 16} {\upchi }_{16 \times 1} = F_{16 \times 1}$$

After solving Eq. (5), the biodynamic responses (STHT and AM) may acquire as,

6 $$STHT = \frac{{Z_{1} \sin \theta_{1} }}{{Z_{0} }}$$

7 $$AM = \frac{{F_{4} }}{{a_{4} }}$$

where *Z*_1_ and *Z*_0_ are vertical displacements at head and seat (input), respectively. *a*_4_ and *F*_4_ are acceleration and force at the contact point between human and seat *i.e.,* pelvis, respectively. *θ*_*1*_ is the backrest angle. For numerical simulation, its value is taken as *θ*_*1*_ = 24°.

STHT is a dimensional less quantity; it helps researchers and designers to investigate the amount and frequency of vibration passes to the human body through a vibrating medium (i.e., seat or floor). Whereas AM provides information about the mass of humans in a dynamic environment. In case of a rigid body AM is the mass of the system in a static state. However, in dynamic conditions at resonance, the apparent mass can quadruple ^16^.

### Model parameters estimation with firefly algorithm

In this section, the biomechanical parameters of the developed model is optimized with the help of the firefly algorithm (FA). The parameters are optimized by reducing the sum square error between the experimental and analytical response. The objective function includes both magnitude and phase responses of STHT and AM as,

8 $${\text{Minimize }}(O_{f} ) = \mathop \Sigma \limits_{i = 1}^{p} (\alpha_{1} .\lambda_{1} + \alpha_{2} .\lambda_{2} + \alpha_{3} .\lambda_{3} + \alpha_{4} .\lambda_{4} )$$

Here

$$\begin{aligned} & \lambda_{1} = [STHT_{E} (f_{i} ) - STHT_{A} (f_{i} )]^{2}_{Mag} {,}\quad \lambda_{2} = [STHT_{E} (f_{i} ) - STHT_{A} (f_{i} )]^{2}_{Pha} \\ & \lambda_{3} = [AM_{E} (f_{i} ) - AM_{A} (f_{i} )]^{2}_{Mag} {,}\quad \, \lambda_{4} = [AM_{E} (f_{i} ) - AM_{A} (f_{i} )]^{2}_{Pha} \\ \end{aligned}$$

**Figure 3:**
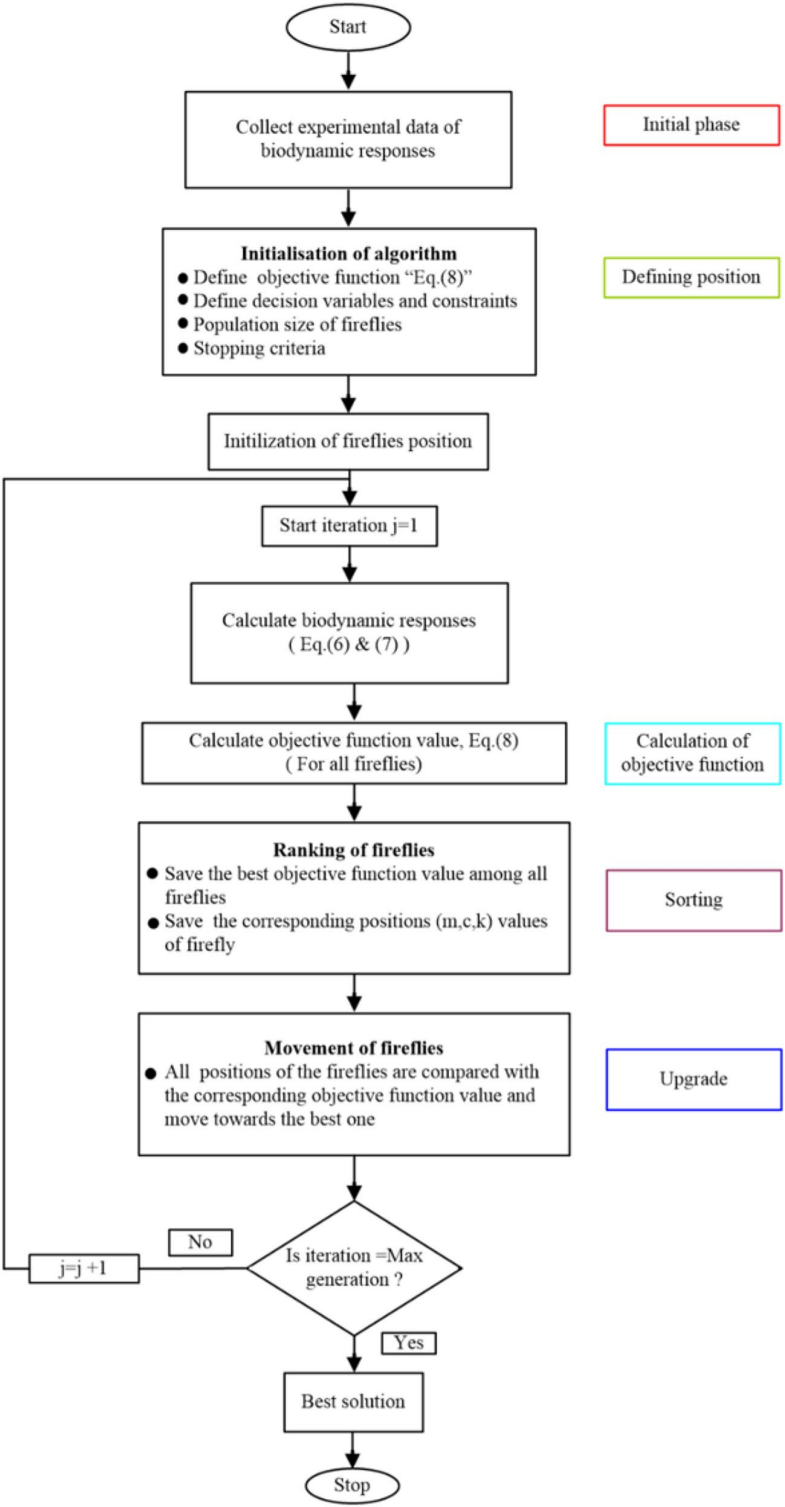
The work flow of firefly algorithm.

The experimental and analytical readings are denoted by the subscripts ‘*E*’ and ‘*A*’, respectively. Subscripts ‘*Mag*’ and ‘*Pha*’ denote magnitude and phase responses, respectively. Symbols *α*_1_,* α*_2_,* α*_3_ are* α*_4_ denote weight functions. Equal importance (weight) is assigned to both the biodynamic responses *i.e.,* (*α*_1_ = *α*_2_ = *α*_3_ = *α*_4_). While ‘*p*’ denotes the number of experimental data points. A flow chart (Fig. 3) represents the typical process followed to optimize the biomechanical parameters. To minimize the objective function and acquire optimized parameters of the human body, the following decision variables and constraints are applied in the analysis.

Decision variables (refer Fig. 2):

1. *m*_1_, *m*_2,_ …*m*_16_ are the segmental mass of the model
2. *K*_1_, *K*_2_, ….*K*_26_ are the stiffness matrices of inbetween segments
3. *C*_1_, *C*_2_, ….*C*_26_ are the damping matrices of inbetween segments

Here direct and cross-coupled stiffness and damping parameters are contained in the stiffness and damping matrices, respectively.

Constraints:

9 $$\left\{ \begin{gathered} \sum\limits_{i = 1}^{16} {m_{i} = 77.3\;{\text{kg}}} \hfill \\ m_{5} = m_{8} ,m_{6} = m_{9} ,m_{7} = m_{10} \hfill \\ m_{11} = m_{14} ,m_{12} = m_{15} ,m_{13} = m_{16} \hfill \\ k_{xz} = k_{zx} ,c_{xz} = c_{zx} \hfill \\ 100\;{\text{Nm}}^{ - 1} < [k_{ii} ] < 300000\;{\text{N}}\;{\text{m}}^{ - 1} \hfill \\ 100\;{\text{Nsm}}^{ - 1} < [c_{ii} ] < 300000\;{\text{N}}\;{\text{s}}\;{\text{m}}^{ - 1} \hfill \\ \end{gathered} \right\}$$

*m*_*i*_, *k*_ij_ and *c*_ij_ are mean weight, lower and upper bounds of stiffness and damping coefficients of the proposed model, respectively. As far as the stiffness values are concerned, the limits are taken from literature^25,26^. They performed a compression test on different segments and drawn the load–deflection curve to obtain the stiffness of different segments. Whereas the damping values are taken from National Institutes of Health (U.S.)^27^. They performed a free vibration test to obtain damping values of each segment. In optimization process the sum squares error (SSE) is calculated to obtain the desired accuracy of optimized parameters. The SSE value was set to 0.000001 to stop the iteration. The other decision parameters selected are total number of variables = 224, swarm size = 100, and the number of iterations = 50. By minimizing the sum square error in Eq. (8) under mentioned constraints in Eq. (9), the model parameters are tuned till the desired accuracy is achieved. The experimental data are referred from^8^ to minimize the objective function. In^8^, the authors conducted experiments on 12 healthy male individuals under random vibration conditions. The magnitude of vibration in the vertical direction is set as 1 ms^−2^ r.m.s in the frequency range of 0.5–15 Hz. The goodness of fit (GOF) is determined as,

10 $$\varepsilon = 1 - \frac{{\sqrt {\Sigma (\tau_{e} - \tau_{a} )^{2} /(N - 2)} }}{{\Sigma \tau_{e} /N}}$$

The experimental and analytical responses are denoted by ‘*τ*_*e*_’ and ‘*τ*_*a*_’ respectively. The total number of data points chosen for analysis is referred to as ‘*N’.* The ‘*ɛ*’ reflects/mimics a good model that might be used instead of an experimental investigation. If the value is 1, it means that the analytical model and experimental responses are identical. It signifies that ‘*ɛ*’ makes the model effectiveness. Table 1 shows the best-optimized parameters for the suggested model with the highest GOF value.

**Table 1:** The optimized mass, stiffness, and damping coefficients with FA.

Mass (kg)									
*m*_1_	*m*_2_	*m*_3_	*m*_4_	*m*_5_	*m*_6_	*m*_7_	*m*_8_	*m*_9_	*m*_10_
6.13	14.39	10.18	11.22	2.26	1.34	0.77	2.26	1.34	0.77
*m*_11_	*m*_12_	*m*_13_	*m*_14_	*m*_15_	*m*_16_	–	–	–	–
8.15	3.14	1.24	8.15	3.14	1.24	–	–	–	–
Stiffness parameters (N m^−1^, × 10^5^)									
$k_{xx}^{1}$	1.62	$k_{xx}^{2}$	1.72	$k_{xx}^{3}$	1.94	$k_{xx}^{4}$	2.24	$k_{xx}^{5}$	1.34
$k_{xz}^{1}$	0.74	$k_{xz}^{2}$	0.49	$k_{xz}^{3}$	0.64	$k_{xz}^{4}$	1.26	$k_{xz}^{5}$	0.61
$k_{zx}^{1}$	0.74	$k_{zx}^{2}$	0.49	$k_{zx}^{3}$	0.64	$k_{zx}^{4}$	1.26	$k_{zx}^{5}$	0.61
$k_{zz}^{1}$	2.39	$k_{zz}^{2}$	2.45	$k_{zz}^{3}$	2.38	$k_{zz}^{4}$	2.51	$k_{zz}^{5}$	1.67
$k_{xx}^{6}$	1.67	$k_{xx}^{7}$	1.17	$k_{xx}^{8}$	1.34	$k_{xx}^{9}$	1.67	$k_{xx}^{10}$	1.17
$k_{xz}^{6}$	0.35	$k_{xz}^{7}$	0.54	$k_{xz}^{8}$	0.61	$k_{xz}^{9}$	0.35	$k_{xz}^{10}$	0.54
$k_{zx}^{6}$	0.35	$k_{zx}^{7}$	0.54	$k_{zx}^{8}$	0.61	$k_{zx}^{9}$	0.35	$k_{zx}^{10}$	0.54
$k_{zz}^{6}$	2.07	$k_{zz}^{7}$	1.85	$k_{zz}^{8}$	1.67	$k_{zz}^{9}$	2.07	$k_{zz}^{10}$	1.85
$k_{xx}^{11}$	1.27	$k_{xx}^{12}$	1.92	$k_{xx}^{13}$	1.72	$k_{xx}^{14}$	1.62	$k_{xx}^{15}$	1.72
$k_{xz}^{11}$	0.85	$k_{xz}^{12}$	1.45	$k_{xz}^{13}$	0.46	$k_{xz}^{14}$	0.42	$k_{xz}^{15}$	0.46
$k_{zx}^{11}$	0.85	$k_{zx}^{12}$	1.45	$k_{zx}^{13}$	0.46	$k_{zx}^{14}$	0.42	$k_{zx}^{15}$	0.46
$k_{zz}^{11}$	1.64	$k_{zz}^{12}$	2.56	$k_{zz}^{13}$	2.19	$k_{zz}^{14}$	1.86	$k_{zz}^{15}$	2.19
$k_{xx}^{16}$	1.62	$k_{xx}^{17}$	1.76	$k_{xx}^{18}$	1.26	$k_{xx}^{19}$	1.09	$k_{xx}^{20}$	1.13
$k_{xz}^{16}$	0.42	$k_{xz}^{17}$	0.34	$k_{xz}^{18}$	0.64	$k_{xz}^{19}$	0.71	$k_{xz}^{20}$	0.32
$k_{zx}^{16}$	0.42	$k_{zx}^{17}$	0.34	$k_{zx}^{18}$	0.64	$k_{zx}^{19}$	0.71	$k_{zx}^{20}$	0.32
$k_{zz}^{16}$	1.86	$k_{zz}^{17}$	2.37	$k_{zz}^{18}$	1.84	$k_{zx}^{19}$	1.76	$k_{zx}^{20}$	1.75
$k_{xx}^{21}$	1.64	$k_{xx}^{22}$	1.76	$k_{xx}^{23}$	1.26	$k_{xx}^{24}$	1.09	$k_{xx}^{25}$	1.13
$k_{xz}^{21}$	0.51	$k_{xz}^{22}$	0.34	$k_{xz}^{23}$	0.64	$k_{xz}^{24}$	0.71	$k_{xz}^{25}$	0.32
$k_{zx}^{21}$	0.51	$k_{zx}^{22}$	0.34	$k_{zx}^{23}$	0.64	$k_{zx}^{24}$	0.71	$k_{zx}^{25}$	0.32
$k_{zz}^{21}$	2.13	$k_{zz}^{22}$	2.37	$k_{zz}^{23}$	1.84	$k_{zz}^{24}$	1.76	$k_{zz}^{25}$	1.75
$k_{zz}^{26}$	1.64	$k_{zz}^{26}$	0.51	$k_{zz}^{26}$	0.51	$k_{zz}^{26}$	2.13		
Damping parameters (N s m^−1^, × 10^3^)									
$c_{xx}^{1}$	1.21	$c_{xx}^{2}$	1.24	$c_{xx}^{3}$	1.06	$c_{xx}^{4}$	1.44	$c_{xx}^{5}$	1.13
$c_{xz}^{1}$	0.45	$c_{xz}^{2}$	0.26	$c_{xz}^{3}$	0.48	$c_{xz}^{4}$	0.67	$c_{xz}^{5}$	0.72
$c_{zx}^{1}$	0.45	$c_{zx}^{2}$	0.26	$c_{zx}^{3}$	0.48	$c_{zx}^{4}$	0.67	$c_{zx}^{5}$	0.72
$c_{zz}^{1}$	2.06	$c_{zz}^{2}$	1.86	$c_{zz}^{3}$	1.26	$c_{zz}^{4}$	2.19	$c_{zz}^{5}$	1.84
$c_{xx}^{6}$	1.34	$c_{xx}^{7}$	1.46	$c_{xx}^{8}$	1.13	$c_{xx}^{9}$	1.34	$c_{xx}^{10}$	1.46
$c_{xz}^{6}$	0.57	$c_{xz}^{7}$	0.61	$c_{xz}^{8}$	0.72	$c_{xz}^{9}$	0.57	$c_{xz}^{10}$	0.61
$c_{zx}^{6}$	0.57	$c_{zx}^{7}$	0.61	$c_{zx}^{8}$	0.72	$c_{zx}^{9}$	0.57	$c_{zx}^{10}$	0.61
$c_{zz}^{6}$	1.94	$c_{zz}^{7}$	2.01	$c_{zz}^{8}$	1.84	$c_{zz}^{9}$	1.94	$c_{zz}^{10}$	2.01
$c_{xx}^{11}$	1.89	$c_{xx}^{12}$	2.26	$c_{xx}^{13}$	1.17	$c_{xx}^{14}$	1.24	$c_{xx}^{15}$	1.17
$c_{xz}^{11}$	1.17	$c_{xz}^{12}$	1.54	$c_{xz}^{13}$	0.84	$c_{xz}^{14}$	0.61	$c_{xz}^{15}$	0.84
$c_{zx}^{11}$	1.17	$c_{zx}^{12}$	1.54	$c_{zx}^{13}$	0.84	$c_{zx}^{14}$	0.61	$c_{zx}^{15}$	0.84
$c_{zz}^{11}$	2.28	$c_{zz}^{12}$	2.61	$c_{zz}^{13}$	2.30	$c_{zz}^{14}$	2.13	$c_{zz}^{15}$	2.30
$c_{xx}^{16}$	1.24	$c_{xx}^{17}$	1.15	$c_{xx}^{18}$	1.26	$c_{xx}^{19}$	1.37	$c_{xx}^{20}$	0.72
$c_{xz}^{16}$	0.61	$c_{xz}^{17}$	0.65	$c_{xz}^{18}$	0.51	$c_{xz}^{19}$	0.65	$c_{xz}^{20}$	0.26
$c_{zx}^{16}$	0.61	$c_{zx}^{17}$	0.65	$c_{zx}^{18}$	0.51	$c_{zx}^{19}$	0.65	$c_{zx}^{20}$	0.26
$c_{zz}^{16}$	2.13	$c_{zz}^{17}$	2.14	$c_{zz}^{18}$	1.96	$c_{zx}^{19}$	2.12	$c_{zx}^{20}$	1.51
$c_{xx}^{21}$	1.15	$c_{xx}^{22}$	1.15	$c_{xx}^{23}$	1.26	$c_{xx}^{24}$	1.37	$c_{xx}^{25}$	0.72
$c_{xz}^{21}$	0.64	$c_{xz}^{22}$	0.65	$c_{xz}^{23}$	0.51	$c_{xz}^{24}$	0.65	$c_{xz}^{25}$	0.26
$c_{zx}^{21}$	0.64	$c_{zx}^{22}$	0.65	$c_{zx}^{23}$	0.51	$c_{zx}^{24}$	0.65	$c_{zx}^{25}$	0.26
$c_{zz}^{21}$	2.14	$c_{zz}^{22}$	2.14	$c_{zz}^{23}$	1.96	$c_{zz}^{24}$	2.12	$c_{zz}^{25}$	1.51
$c_{zz}^{26}$	1.15	$c_{zz}^{26}$	0.64	$c_{zz}^{26}$	0.64	$c_{zz}^{26}$	2.14		

### Biodynamic responses

**Figure 4:**
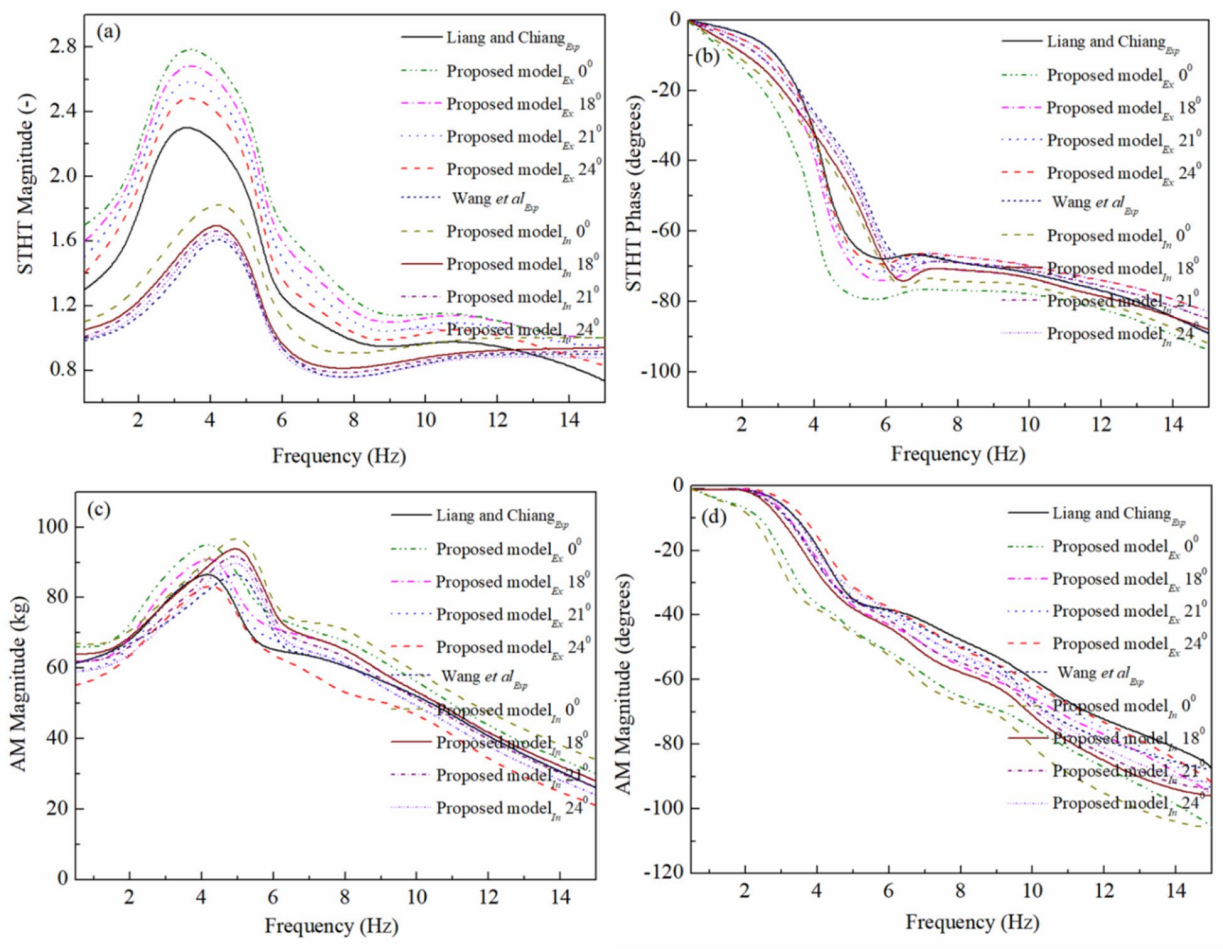
The seat-to-head transmissibility and apparent mass values for seated occupants under random vibration.

The biodynamic responses (magnitude and phase) of STHT and AM are depicted in Fig. 4. For automotive passengers, the authors in^28^ proposed comfortable backrest inclination angles as 18°, 21° and 24°. In the present article, along with these three angles, the vertical backrest (*θ* = 0°) is added to the numerical analysis and compared with the experimental biodynamic responses. In Fig. 4, the biodynamic responses are plotted with and without inclusion of thighs, legs and feet. Also, these responses are compared with the experimental study to visualize the inclusion effect of lower limbs. The subscripts ‘ex’ and ‘in’ used with legends in Fig. 4 represents excluding and including lower limbs, respectively. From Fig. 4, the effect of lower limbs (thighs, legs and foot) on both biodynamic responses may be observed. The primary resonance frequency for the seat to head transmissibility is around 4 Hz when excluding and 5 Hz when including lower limbs. The similar phenomenon may be observed for the apparent mass response.

Figure 4 represents the good agreement between the experimental response and the proposed model with 21° backrest angle both in magnitude and phase. Some deviation in the responses may observe particularly at high frequencies (> 12 Hz). From Fig. 4 it may notice that both STHT and AM responses are deviating more at vertical backrest (*θ* = 0°) conditions as compared to inclined backrest conditions. The obtained overall goodness of fit (GOF) value is (ε = 95.10%) for the back rest angle *θ*_1_ = 24°. Also, the deviation between the experimental response and the simulated response at different back rest angles are obtained as 8.14%, 5.62%, 4.68% and 4.90% for 0°, 18°, 21° and 24° backrest angles, respectively. The deviation is calculated with the following formula: δ = (100 − ε)%, ‘δ’ is the deviation, ‘ε’ is the goodness of fit.

## Sensitivity analysis

The human body is a highly organized and complicated system made up of various organs that works together to accomplish a certain activity. Since, the brain, which is a vital component of the head, connects the human organs and sensory systems. The vibration coming on to the head has been given prime importance and corrective measures have been taken to reduce the amount of vibration passing to the head^29^. Hence, the influence of magnitude variation in characteristic parameters on the biodynamic response (STHT) is performed in this section. One-factor-at-a-time (OAT) technique is used to accomplish sensitivity analysis^30^. As OAT technique is employed, one parameter is deliberately assigned ± 20% deviation from the optimum value keeping the rest of the parameters constant to investigate the influence of deviation on STHT. The influence of all other parameters is determined using the same technique.The ‘+’ and ‘−’ sign implies an increase and decrease in magnitude, respectively. The mathematical expression for OAT in a positive direction may represent as,

11 $$\Delta_{i}^{ + } z = \frac{{f(y_{i}^{ + } ) - f(y_{i}^{0} )}}{{f(y^{0} )}}$$

**Figure 5:**
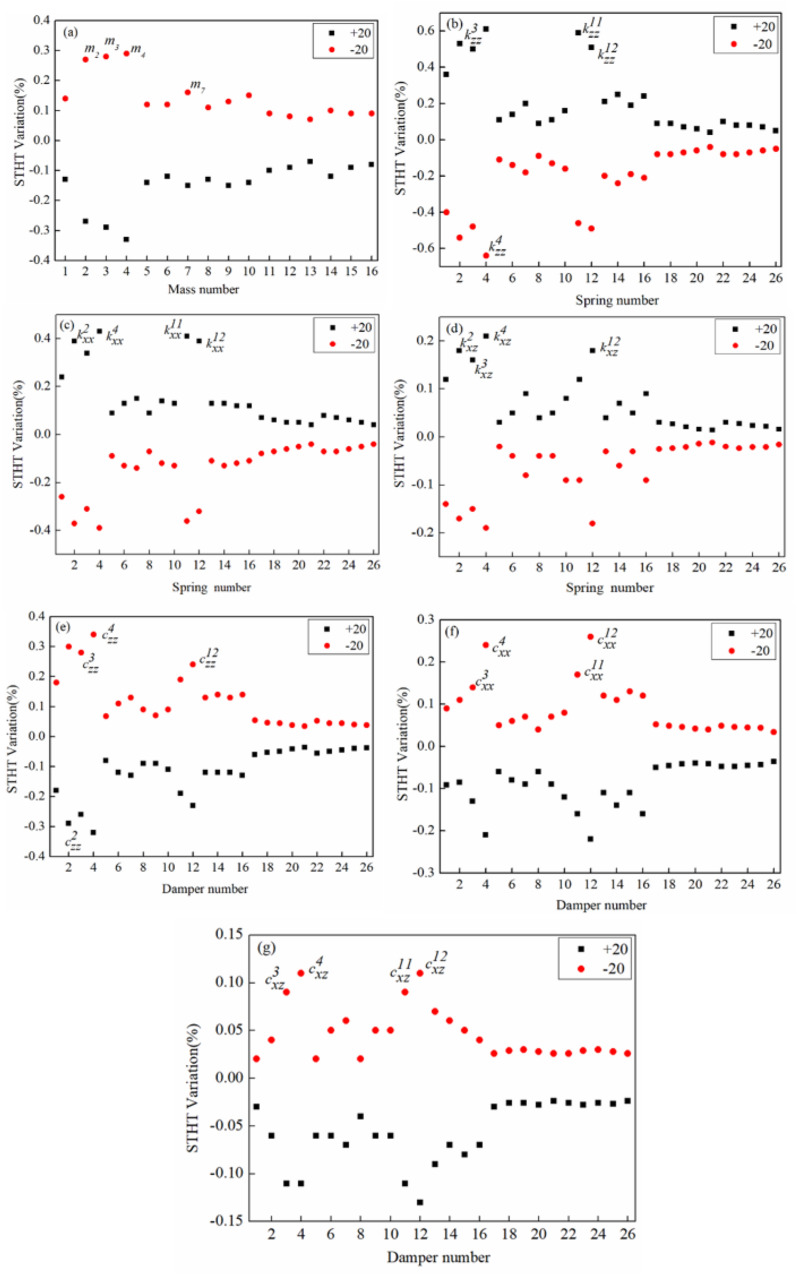
Variation in STHT with direct and cross coupled optimized parameters (mass, stiffness and damping).

Figure 5 depicts the influence on STHT peak value due to magnitude variation (‘+’ and ‘−’) in optimized characteristic parameters. Figure 5a demonstrates the influence of magnitude variation in mass parameters. The absolute deviation in STHT peak value is comparable due to positive and negative magnitude variation in mass parameters. In addition, it may observe that pelvic mass (*m*_4_) and abdomen mass (*m*_3_) are the two most sensitive parameters. Figure 5b–d shows the percentage deviation in STHT peak value for direct stiffness parameters in the vertical, fore-and-aft, and cross-coupled stiffness parameters, respectively. In comparison to cross-coupled stiffness parameters, most direct stiffness parameters in the vertical plane have a considerable influence on STHT, as shown in Fig. 5b–d. It is also worth noting that direct vertical stiffness at the pelvis location ($k_{zz}^{{4}}$) and lumber support ($k_{zz}^{{{11}}} ,k_{zz}^{{{12}}}$) are the most influential parameters. Figure 5e–g depicts the effect of magnitude variation (± 20%) of direct damping parameters in the vertical and fore-and-aft directions, as well as cross-coupled damping parameters, on STHT, respectively.

Alike stiffness parameters, direct damping parameters have greater influence in comparison with cross-coupled parameters on STHT. Figure 5e–g indicates that direct damping in a vertical direction at pelvis & lumber support locations ($c_{zz}^{{4}}$,$k_{zz}^{{{11}}} ,k_{zz}^{{{12}}}$) has a high influence on STHT. Comparing Fig. 5a–g witnessed optimized parameters at the pelvis and lumber locations are the highely sensitive. In addition, it is evident that vertical parameters are more sensitive and have a high influence on STHT in comparison with fore and aft and cross-coupling characteristics. The findings suggest that design engineers must pay attention to the pelvis and lumber support locations to improve human health, safety, and ride comfort.

## Segmental vibration transmissibility (SVT)

The human body is a complex physical and biological system consisting of critical organs. Each organ has its own inherent limiting frequency under which it performs precisely. The cognizance of limiting frequency and dynamic response can help to reduce the adverse effects of vibration on health, comfort, and activities. In the past, researchers were more concerned about the transmissibility ratio (TR) between the seat (input) and the human head (output) known as STHT, but the segmental transmissibility analysis is as important as STHT to understand the adverse effect of input vibration on each critical organs/parts. To enrich the cognizance regarding SVT, the vibration transmissibility from the seat to the head, thorax, abdomen, and pelvis are analyzed. The TR may mathematically expressed as,

12 $$TR = \frac{{z_{o} (j\omega )}}{{z_{i} (j\omega )}}$$

Here *z*_*o*_ (*jɷ*) is the Fourier transform of displacement amplitude at the desired segment/organ considered as output and *z*_*i*_ (*jɷ*) is Fourier transform of displacement amplitude from the seat considered as input.

**Figure 6:**
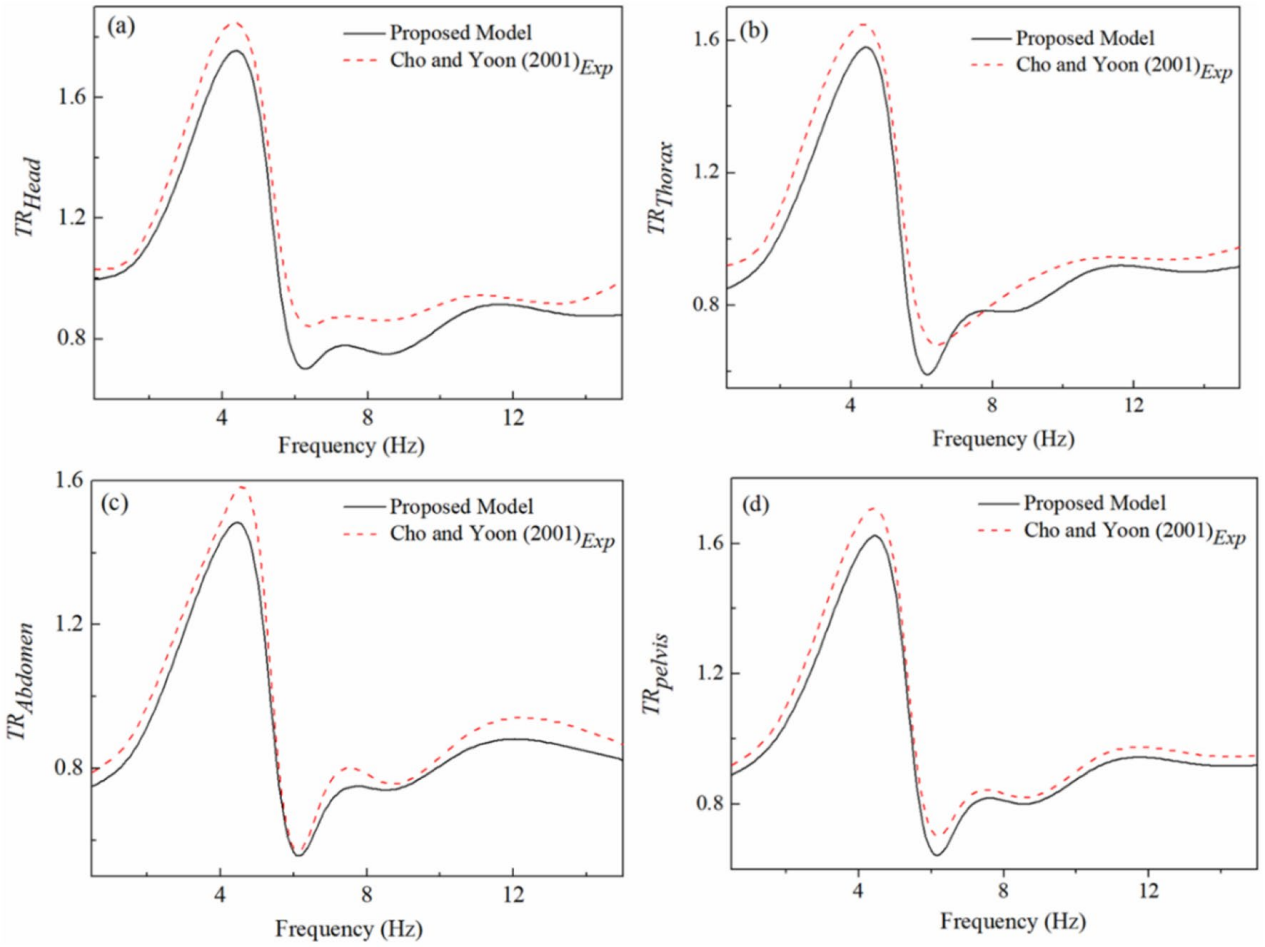
Transmissibility ratio at critical segments.

The Eq. (12) is utilized to acquire the transmissibility ratio among seat-to-head (*Z*_*1*_*/Z*_*0*_), seat-to-thorax (*Z*_*2*_*/Z*_*0*_), seat-to-abdomen (*Z*_*3*_*/Z*_*0*_), and seat-to-pelvis (*Z*_*4*_*/Z*_*0*_) of the seated occupant. Figure 6 represents the variation in SVT with frequency. From Fig. 6 it may observe that all the critical organs (head, thorax, abdomen, and pelvis) represent maximum vibration transmissibility between 4 and 6 Hz. Upon comparison Fig. 6a–d it may observe that the input vibration has maximum influence on the head and least on the abdomen at the resonance frequency. As head is not having any support, it may be considered as having flexible connection with human body. Hence the head segment attained the maximum transmissibility in comparison with the other segments (thorax, abdomen and pelvis). The outcomes are helpful to design engineers to monitor the segmental transmissibility in seated occupants. In addition, it will serve as a tool to assess the requirement of protective measures with fewer or no human tests.

## Ride comfort analysis

**Figure 7:**
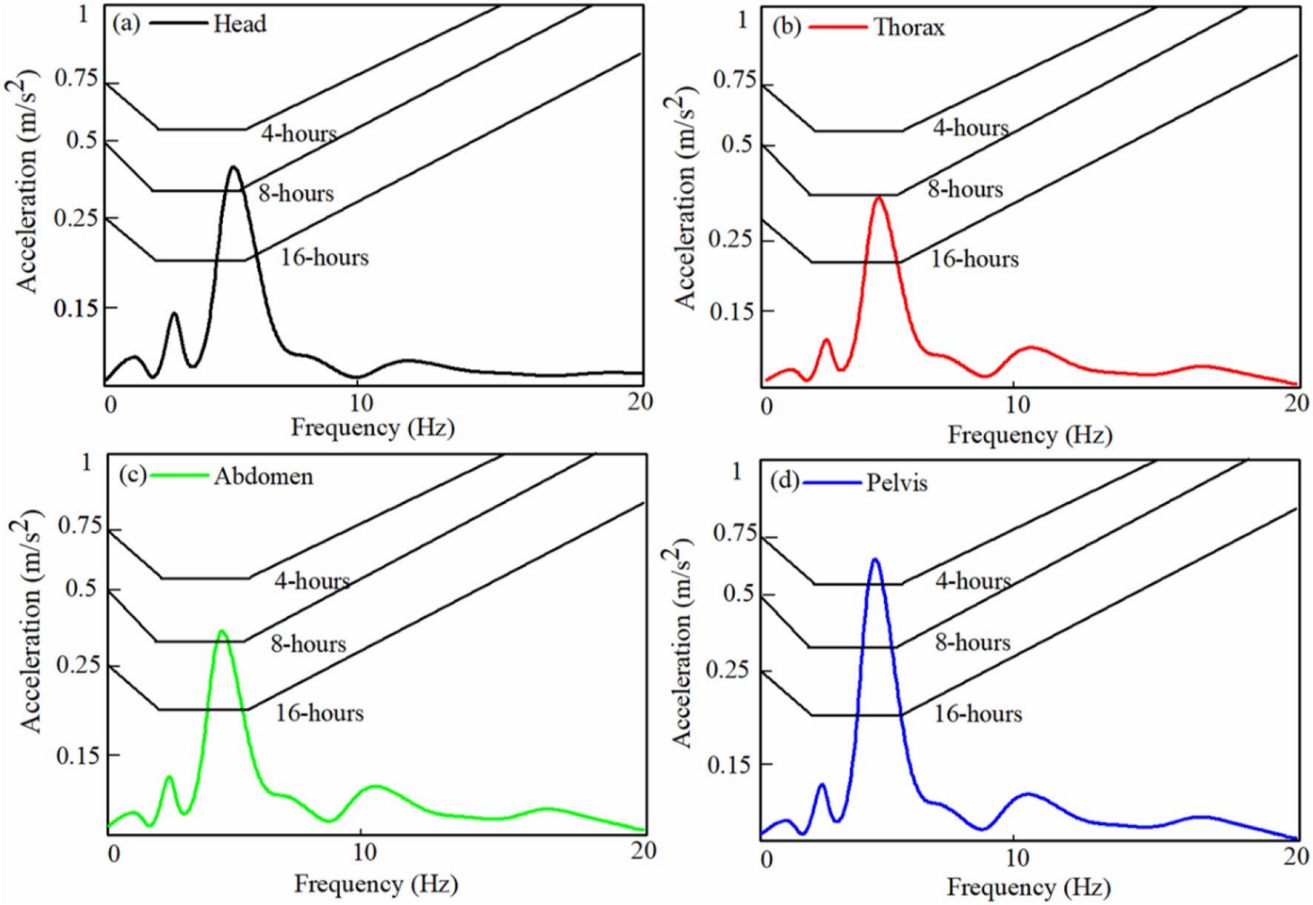
Variation in acceleration (**a**) head (**b**) thorax (**c**) abdomen (**d**) pelvis with the ISO 2631-1:1997 curves.

In-vehicle dynamics, vibration is the most influencing parameter to ride comfort in seated occupants. When vibration in a vehicle exceeds a specific threshold value, the occupants experience discomfort. In past, several methods have been proposed to estimate vehicle ride comfort that includes International Standard Organization (ISO-2631), British Standards (BS-6841), and European standards EN-12, 299^31^. However, the majority use the regulations issued by the International Standard Organization (ISO-2631) to measure the effects of vibration on humans. The ride comfort study is carried out in this section when the developed 32-*dofs* seated person model is combined with a 7-*dofs* full automobile model (see Table A1)^32^. To perform ride comfort analysis, harmonic excitations (*z* = *Ze*^*jωt*^) with an amplitude (*Z* = 0.025 m) at four wheels are given as input. The coupled equation of motion of humans with a full car model derived by^18^ is modified with 32-*dofs* seated occupant model. To acquire the acceleration response of various segments (head, thorax, abdomen, and pelvis) a code is written on MATLAB. The acquired responses are compared to the ISO 2631-1: 1997 ride comfort charts Fig. 7. In Fig. 7, 4-h, 8-h, and 16-h curves indicate the threshold value of vibration exposure for ride comfort. From Fig. 7 it may notice that the peak value of the transmitted vibration to the human body is more sensitive in the frequency range of 5–10 Hz. Further, it may observe that passengers exposed to harmonic excitations experience discomfort at head, thorax and abdomen location after 8-h of exposure and at the pelvis location after 4-h of exposure. Overall, it may conclude that after 4–8 h of exposure under harmonic excitations in the range 5–10 Hz, the seated occupants start experiencing discomfort. Hence, to avoid ride discomfort the human being should not expose to vibration for more than 8-h under harmonic excitation condition. Furthermore it may observe that the vibration magnitude at pelvis is more in comparison with the other segments because the pelvis is in the direct contact with the seat whereas the other segments are away from the seat. Hence it may be considered as the pelvis and the seat are rigidly connected. Whereas the other segments (head, thorax and abdomen) are flexibly connected due to number of in-between segments.

## Conclusions

Seated occupants encounter performance deficiency, MSDS and ride discomfort due to whole-body vibration. The challenges to automobile designers are to estimate the dynamic response of human being accurately under vibrating medium to enhance ride comfort and performance time. A 32-*dofs* seated occupant model coupled with 7-*dofs* full car model is proposed in this article. The influence of magnitude variation in model parameters (± 20% deviation in mass, stiffness and damping) on STHT is investigated. In addition, SVT is analyzed to obtain the most influenced organ under vertical vibration conditions. Simulated biodynamic responses (STHT and AM) are compared with experimental readings to obtain the best ride comfort position. Additionally, the ride comfort analysis for critical organs is performed and compared with the ISO 2631-1:1997 charts. The significant outcomes of this research are:

1. Developed an algorithm to acquire optimized model parameters (mass, stiffness and damping) of seated occupants.
2. Analyzed biodynamic responses (STHT and AM) and compared with experimental readings with overall goodness of fit value as 95.10%.
3. Direct model parameters $(k_{zz}^{{4}} ,c_{zz}^{{4}} ,k_{zz}^{{{11}}} {\text{ and }}k_{zz}^{{{12}}} )$ are obtained as the most influencing parameters having a significant effect on STHT. Whereas, fore-and-aft and cross-coupling parameters have the least influence on STHT.
4. SVT analysis shows seat-to-head transmissibility is more as compared with other critical organs.
5. SVT analysis depicts all the critical organs show a maximum deviation between 4 and 6 Hz.
6. According to ride comfort study, it was noticed that pelvis is the most sensitive organ that experiences discomfort after 4-h of harmonic excitation exposure,

## Supplementary Information

Supplementary Information.

## Data Availability

The datasets used during the current study are available from the corresponding author on reasonable request.
