# Supplementary material for: Ride comfort and segmental vibration transmissibility analysis of an automobile passenger model under whole body vibration
Source: Sci Rep. 2023 Jul 18;13:11619. doi: 10.1038/s41598-023-38592-x (PMC10354197; doi:10.1038/s41598-023-38592-x)
Supplement: Supplementary file 1 — Supplementary Information. [file 41598_2023_38592_MOESM1_ESM.pdf]

Appendix A: Derivation of equation of motion of coupled vehicle-passenger model

Elements of mass matrix $[M]$ (A.1)

$$M_{1,1}=M_{2,2}=m_1;\ M_{3,3}=M_{4,4}=m_2;\ M_{5,5}=m_3\cos\theta_1;\ M_{6,6}=m_3\sin\theta_1;$$

$$M_{7,7}=m_4\cos\theta_1;\ M_{8,8}=m_4\sin\theta_1;\ M_{9,9}=M_{10,10}=m_5;\ M_{11,11}=M_{12,12}=m_6;$$

$$M_{13,13}=M_{14,14}=m_7;\ M_{15,15}=M_{16,16}=m_8;\ M_{17,17}=M_{18,18}=m_9;\ M_{19,19}=M_{20,20}=m_{10};$$

Elements of stiffness matrix $[K]$ (A.2)

$$K_{1,1}=k_{xx}^1;\ K_{1,2}=k_{xz}^1;\ K_{1,3}=K_{3,1}=-k_{xx}^1;\ K_{1,4}=K_{3,2}=-k_{xz}^1;\ K_{2,1}=k_{zx}^1;\ K_{2,2}=k_{zz}^1;$$

$$K_{2,3}=K_{4,1}=-k_{zx}^1;\ K_{2,4}=K_{4,2}=-k_{zz}^1;\ K_{3,3}=k_{xx}^1+k_{xx}^2+k_{xx}^5+k_{xx}^8;\ K_{3,4}=k_{xz}^1+k_{xz}^2+k_{xz}^5+k_{xz}^8;$$

$$K_{3,5}=K_{5,3}=-k_{xx}^2;\ K_{3,6}=K_{5,4}=-k_{xz}^2;\ K_{3,9}=K_{9,3}=-k_{xx}^5;\ K_{3,10}=K_{9,4}=-k_{xz}^5;\ K_{3,15}=-k_{xx}^8;$$

$$K_{3,16}=-k_{xz}^8;\ K_{4,3}=k_{zx}^1+k_{zx}^2+k_{zx}^5+k_{zx}^8;\ K_{4,4}=k_{zz}^1+k_{zz}^2+k_{zz}^5+k_{zz}^8;\ K_{4,5}=K_{6,3}=-k_{zx}^2;$$

$$K_{4,6}=K_{6,4}=-k_{zz}^2;\ K_{4,9}=K_{10,3}=-k_{zx}^5;\ K_{4,10}=K_{10,10}=-k_{zz}^5;\ K_{4,15}=-k_{zx}^8;\ K_{4,16}=-k_{zz}^8;$$

$$K_{5,5}=k_{xx}^{11}\cos\theta_1+k_{xx}^2+k_{xx}^3;\ K_{5,6}=k_{xz}^{11}\cos\theta_1+k_{xz}^2+k_{xz}^3;\ K_{5,7}=K_{7,5}=-k_{xx}^3;\ K_{5,8}=K_{7,6}=-k_{xz}^3;$$

$$K_{6,5}=k_{zx}^{11}\sin\theta_1+k_{zx}^2+k_{zx}^3;\ K_{6,6}=k_{zz}^{11}\sin\theta_1+k_{zz}^2+k_{zz}^3;\ K_{6,7}=K_{8,5}=-k_{zx}^3;\ K_{6,8}=K_{8,6}=-k_{zz}^3;$$

$$K_{7,7}=k_{xx}^{12}\cos\theta_1+k_{xx}^3+k_{xx}^4;\ K_{7,8}=k_{xz}^{12}\cos\theta_1+k_{xz}^3+k_{xz}^4;\ K_{8,7}=k_{zx}^{12}\sin\theta_1+k_{zx}^3+k_{zx}^4;$$

$$K_{8,8}=k_{zz}^{12}\sin\theta_1+k_{zz}^3+k_{zz}^4;\ K_{9,9}=k_{xx}^5+k_{xx}^6;\ K_{9,10}=k_{xz}^5+k_{xz}^6;\ K_{9,11}=K_{11,9}=-k_{xx}^6;$$

$$K_{9,12}=K_{11,10}=-k_{xz}^6;\ K_{10,9}=k_{zx}^5+k_{zx}^6;\ K_{10,10}=k_{zz}^5+k_{zz}^6;\ K_{10,11}=K_{12,9}=-k_{zx}^6;$$

$$K_{10,12}=K_{12,10}=-k_{zz}^6;\ K_{11,11}=k_{xx}^{13}+k_{xx}^6+k_{xx}^7;\ K_{11,12}=k_{xz}^{13}+k_{xz}^6+k_{xz}^7;\ K_{11,13}=K_{13,11}=-k_{xx}^7;$$

$$K_{11,14}=K_{13,12}=-k_{xz}^7;\ K_{12,11}=k_{zx}^{13}+k_{zx}^6+k_{zx}^7;\ K_{12,12}=k_{zz}^{13}+k_{zz}^6+k_{zz}^7;\ K_{12,13}=K_{14,11}=-k_{zx}^7;$$

$$K_{12,14}=K_{14,12}=-k_{zz}^7;\ K_{13,13}=k_{xx}^7+k_{xx}^{14};\ K_{13,14}=k_{xz}^7+k_{xz}^{14};\ K_{14,13}=k_{zx}^7+k_{zx}^{14};\ K_{14,14}=k_{zz}^7+k_{zz}^{14};$$

$$K_{15,3}=-k_{xx}^{18};\ K_{15,4}=-k_{xz}^{18};\ K_{15,15}=k_{xx}^8+k_{xx}^9;\ K_{15,16}=k_{xz}^8+k_{xz}^9;\ K_{15,17}=K_{17,15}=-k_{xx}^9;$$

$$K_{15,18}=K_{17,16}=-k_{xz}^9;\ K_{16,3}=-k_{zx}^{18};\ K_{16,4}=-k_{zz}^{18};\ K_{16,15}=k_{zx}^8+k_{zx}^9;\ K_{16,16}=k_{zz}^8+k_{zz}^9;$$

$$K_{16,17}=K_{18,15}=-k_{zx}^9;\ K_{16,18}=K_{18,16}=-k_{zz}^9;\ K_{17,17}=k_{xx}^9+k_{xx}^{15}+k_{xx}^{10};\ K_{17,18}=k_{xz}^9+k_{xz}^{15}+k_{xz}^{10};$$

$$K_{17,19}=K_{19,17}=-k_{xx}^{10};\ K_{17,20}=K_{19,18}=-k_{xz}^{10};\ K_{18,17}=k_{zx}^9+k_{zx}^{15}+k_{zx}^{10};\ K_{18,18}=k_{zz}^9+k_{zz}^{15}+k_{zz}^{10};$$

$K_{18,19}=K_{20,17}=-k_{zx}^{10};\ K_{18,20}=K_{20,18}=-k_{zz}^{10};\ K_{19,19}=k_{xx}^{10}+k_{xx}^{16};\ K_{19,20}=k_{xz}^{10}+k_{xz}^{16};$

$K_{20,19}=k_{zx}^{10}+k_{zx}^{16};\ K_{20,20}=k_{zz}^{10}+k_{zz}^{16};$

Elements of damping matrix $[C]$ (A.3)

$C_{1,1}=c_{xx}^{1};\ C_{1,2}=c_{xz}^{1};\ C_{1,3}=C_{3,1}=-c_{xx}^{1};\ C_{1,4}=C_{3,2}=-c_{xz}^{1};\ C_{2,2}=c_{zz}^{1};\ C_{2,3}=C_{4,1}=-c_{zx}^{1};$

$C_{2,4}=C_{4,2}=-c_{zz}^{1};\ C_{3,3}=c_{xx}^{1}+c_{xx}^{2}+c_{xx}^{5}+c_{xx}^{8};\ C_{3,4}=c_{xz}^{1}+c_{xz}^{2}+c_{xz}^{5}+c_{xz}^{8};\ C_{3,5}=C_{5,3}=-c_{xx}^{2};$

$C_{3,6}=C_{5,4}=-c_{xz}^{2};\ C_{3,9}=C_{9,3}=-c_{xx}^{5};\ C_{3,10}=C_{9,4}=-c_{xz}^{5};\ C_{3,15}=-c_{xx}^{8};\ C_{3,16}=-c_{xz}^{8};$

$C_{4,3}=c_{zx}^{1}+c_{zx}^{2}+c_{zx}^{5}+c_{zx}^{8};\ C_{4,4}=c_{zz}^{1}+c_{zz}^{2}+c_{zz}^{5}+c_{zz}^{8};\ C_{4,5}=C_{6,3}=-c_{zx}^{2};\ C_{4,6}=C_{6,4}=-c_{zz}^{2};$

$C_{4,9}=C_{10,3}=-c_{zx}^{5};\ C_{4,10}=C_{10,10}=-c_{zz}^{5};\ C_{4,15}=-c_{zx}^{8};\ C_{4,16}=-c_{zz}^{8};\ C_{5,5}=c_{xx}^{11}\cos\theta_1+c_{xx}^{2}+c_{xx}^{3};$

$C_{5,6}=c_{xz}^{11}\cos\theta_1+c_{xz}^{2}+c_{xz}^{3};\ C_{5,7}=C_{7,5}=-c_{xx}^{3};\ C_{5,8}=C_{7,6}=-c_{xz}^{3};\ C_{6,5}=c_{zx}^{11}\sin\theta_1+c_{zx}^{2}+c_{zx}^{3};$

$C_{6,6}=c_{zz}^{11}\sin\theta_1+c_{zz}^{2}+c_{zz}^{3};\ C_{6,7}=C_{8,5}=-c_{zx}^{3};\ C_{6,8}=C_{8,6}=-c_{zz}^{3};\ C_{7,7}=c_{xx}^{12}\cos\theta_1+c_{xx}^{3}+c_{xx}^{4};$

$C_{7,8}=c_{xz}^{12}\cos\theta_1+c_{xz}^{3}+c_{xz}^{4};\ C_{8,7}=c_{zx}^{12}\sin\theta_1+c_{zx}^{3}+c_{zx}^{4};\ C_{8,8}=c_{zz}^{12}\sin\theta_1+c_{zz}^{3}+c_{zz}^{4};$

$C_{9,10}=c_{xz}^{5}+c_{xz}^{6};\ C_{9,11}=C_{11,9}=-c_{xx}^{6};\ C_{9,12}=C_{11,10}=-c_{xz}^{6};\ C_{10,9}=c_{zx}^{5}+c_{zx}^{6};\ C_{10,10}=c_{zz}^{5}+c_{zz}^{6};$

$C_{10,11}=C_{12,9}=-c_{zx}^{6};\ C_{10,12}=C_{12,10}=-c_{zz}^{6};\ C_{11,11}=c_{xx}^{13}+c_{xx}^{6}+c_{xx}^{7};\ C_{11,12}=c_{xz}^{13}+c_{xz}^{6}+c_{xz}^{7};$

$C_{11,13}=C_{13,12}=-c_{xx}^{7};\ C_{11,14}=C_{13,12}=-c_{xz}^{7};\ C_{12,11}=c_{zx}^{13}+c_{zx}^{6}+c_{zx}^{7};\ C_{12,12}=c_{zz}^{13}+c_{zz}^{6}+c_{zz}^{7};$

$C_{12,13}=C_{14,11}=-c_{zx}^{7};\ C_{12,14}=C_{14,12}=-c_{zz}^{7};\ C_{13,13}=c_{xx}^{7}+c_{xx}^{14};\ C_{13,14}=c_{xz}^{7}+c_{xz}^{14};\ C_{14,13}=c_{zx}^{7}+c_{zx}^{14};$

$C_{14,14}=c_{zz}^{7}+c_{zz}^{14};\ C_{15,3}=-c_{xx}^{18};\ C_{15,4}=-c_{xz}^{18};\ C_{15,15}=c_{xx}^{8}+c_{xx}^{9};\ C_{15,16}=c_{xz}^{8}+c_{xz}^{9};\ C_{15,17}=C_{17,15}=-c_{xx}^{9};$

$C_{15,18}=C_{17,16}=-c_{xz}^{9};\ C_{16,3}=-c_{zx}^{18};\ C_{16,4}=-c_{zz}^{18};\ C_{16,15}=c_{zx}^{8}+c_{zx}^{9};\ C_{16,16}=c_{zz}^{8}+c_{zz}^{9};$

$C_{16,17}=C_{18,15}=-c_{zx}^{9};\ C_{16,18}=C_{18,16}=-c_{zz}^{9};\ C_{17,17}=c_{xx}^{9}+c_{xx}^{15}+c_{xx}^{10};\ C_{17,18}=c_{xz}^{9}+c_{xz}^{15}+c_{xz}^{10};$

$C_{17,19}=C_{19,17}=-c_{xx}^{10};\ C_{17,20}=C_{19,18}=-c_{xz}^{10};\ C_{18,17}=c_{zx}^{9}+c_{zx}^{15}+c_{zx}^{10};\ C_{18,18}=c_{zz}^{9}+c_{zz}^{15}+c_{zz}^{10};$

$C_{18,19}=C_{20,17}=-c_{zx}^{10};\ C_{18,20}=C_{20,18}=-c_{zz}^{10};\ C_{19,19}=c_{xx}^{10}+c_{xx}^{16};\ C_{19,20}=c_{xz}^{10}+c_{xz}^{16};\ C_{20,19}=c_{zx}^{10}+c_{zx}^{16};$

$C_{20,20}=c_{zz}^{10}+c_{zz}^{16};$

Elements of force vector $\{f\}$ (A.4)

$f_{5,2}=\{k_{xz}^{11}\cos\theta_1\}z_0+\{c_{xz}^{11}\cos\theta_1\}\dot{z}_0;\ f_{6,2}=\{k_{zz}^{11}\sin\theta_1\}z_0+\{c_{zz}^{11}\sin\theta_1\}\dot{z}_0;$

$$f_{7,2}=\{k_{xz}^{4}+k_{xz}^{12}\cos\theta_1\}z_0+\{c_{xz}^{4}+c_{xz}^{12}\cos\theta_1+\}\dot{z}_0;\; f_{8,2}=\{k_{zz}^{4}+k_{zz}^{12}\sin\theta_1\}z_0+\{c_{zz}^{4}+c_{zz}^{12}\sin\theta_1\}\dot{z}_0;$$

$$f_{12,1}=\{k_{xz}^{13}+k_{zz}^{13}\}z_0+\{c_{xz}^{13}+c_{zz}^{13}\}\dot{z}_0;\; f_{13,2}=\{k_{xz}^{14}\}z_0+\{c_{xz}^{14}\}\dot{z}_0;\; f_{14,2}=\{k_{zz}^{14}\}z_0+\{c_{zz}^{14}\}\dot{z}_0;$$

$$f_{17,2}=\{k_{xz}^{15}\}z_0+\{c_{xz}^{15}\}\dot{z}_0;\; f_{18,2}=\{k_{zz}^{15}\}z_0+\{c_{zz}^{15}\}\dot{z}_0;\; f_{19,2}=\{k_{xz}^{16}\}z_0+\{c_{xz}^{16}\}\dot{z}_0;$$

$$f_{20,2}=\{k_{zz}^{16}\}z_0+\{c_{zz}^{16}\}\dot{z}_0;$$

Rest of the elements of the matrices are zero.

The equation of motion for chassis and seat are as follows:

$$\begin{aligned}&m_c\ddot{z}_c+k_{w_2}(z_c-L_1\theta+L_3\phi-z_{w_2})+c_{w_2}(\dot{z}_c-L_1\dot{\theta}+L_3\dot{\phi}-\dot{z}_{w_2})+k_{w_4}(z_c+L_2\theta+L_3\phi-z_{w_4})+\\&c_{w_4}(\dot{z}_c+L_2\dot{\theta}+L_3\dot{\phi}-\dot{z}_{w_4})+k_{w_1}(z_c-L_1\theta-L_3\phi-z_{w_1})+c_{w_1}(\dot{z}_c-L_1\dot{\theta}-L_3\dot{\phi}-\dot{z}_{w_1})+\\&k_{w_3}(z_c+L_2\theta-L_3\phi-z_{w_3})+c_{w_3}(\dot{z}_c+L_2\dot{\theta}-L_3\dot{\phi}-\dot{z}_{w_3})-k_s(z_s-z_c-r_x\theta-r_y\phi)\\&-c_s(\dot{z}_s-\dot{z}_c-r_x\dot{\theta}-r_y\dot{\phi})=0\end{aligned}\tag{A.5}$$

$$\begin{aligned}&I_{xx}\ddot{\phi}+L_3k_{w_2}(z_c-L_1\theta+L_3\phi-z_{w_2})-L_3c_{w_2}(\dot{z}_c-L_1\dot{\theta}+L_3\dot{\phi}-\dot{z}_{w_2})+L_3k_{w_4}(z_c+L_2\theta+L_3\phi-z_{w_4})+\\&L_3c_{w_4}(\dot{z}_c+L_2\dot{\theta}+L_3\dot{\phi}-\dot{z}_{w_4})-L_3k_{w_1}(z_c-L_1\theta+L_3\phi-z_{w_1})-L_3c_{w_1}(\dot{z}_c-L_1\dot{\theta}+L_3\dot{\phi}-\dot{z}_{w_1})+\\&L_3k_{w_3}(z_c+L_1\theta-L_3\phi-z_{w_3})-L_3c_{w_3}(\dot{z}_c+L_1\dot{\theta}-L_3\dot{\phi}-\dot{z}_{w_3})+r_yk_s(z_s-z_c-r_x\theta-r_y\phi)\\&-r_yc_s(\dot{z}_s-\dot{z}_c-r_x\dot{\theta}-r_y\dot{\phi})=0\end{aligned}\tag{A.6}$$

$$\begin{aligned}&I_{yy}\ddot{\phi}-L_1k_{w_2}(z_c-L_1\theta+L_3\phi-z_{w_2})+L_1c_{w_2}(\dot{z}_c-L_1\dot{\theta}+L_3\dot{\phi}-\dot{z}_{w_2})+L_2k_{w_4}(z_c+L_2\theta+L_3\phi-z_{w_4})+\\&L_2c_{w_4}(\dot{z}_c+L_2\dot{\theta}+L_3\dot{\phi}-\dot{z}_{w_4})-L_1k_{w_1}(z_c-L_1\theta-L_3\phi-z_{w_1})-L_1c_{w_1}(\dot{z}_c-L_1\dot{\theta}-L_3\dot{\phi}-\dot{z}_{w_1})+\\&L_3k_{w_3}(z_c+L_2\theta-L_3\phi-z_{w_3})+L_3c_{w_3}(\dot{z}_c+L_2\dot{\theta}-L_3\dot{\phi}-\dot{z}_{w_3})+r_xk_s(z_s-z_c-r_x\theta-r_y\phi)\\&+r_xc_s(\dot{z}_s-\dot{z}_c-r_x\dot{\theta}-r_y\dot{\phi})=0\end{aligned}\tag{A.7}$$

$$\begin{aligned}&m_s\ddot{z}_s+k_s(z_s-z_c-r_x\theta-r_y\phi)+c_s(\dot{z}_s-\dot{z}_c-r_x\dot{\theta}-r_y\dot{\phi})-k_{zz}^{4}(z_s-z_4)-c_{zz}^{4}(\dot{z}_s-\dot{z}_4)\\&-k_{zz}^{12}\sin\theta_1(z_s-z_4)-c_{zz}^{12}(\dot{z}_s-\dot{z}_4)-k_{zz}^{6}(z_s-z_6)-c_{zz}^{6}(\dot{z}_s-\dot{z}_6)-k_{zz}^{11}(z_s-z_3)-c_{zz}^{11}(\dot{z}_s-\dot{z}_3)\\&-k_{zz}^{7}(z_s-z_7)-c_{zz}^{7}(\dot{z}_s-\dot{z}_7)-k_{zz}^{9}(z_s-z_9)-c_{zz}^{9}(\dot{z}_s-\dot{z}_9)-k_{zz}^{10}(z_s-z_{10})-c_{zz}^{10}(\dot{z}_s-\dot{z}_{10})=0\end{aligned}\tag{A.8}$$

Table A1. Specifications of Full car model (Ahmed, 2001)

Segments symbol	Segments description	Value (units)
m_c	Chassis mass	679.2 (kg)
m_{w_1}	Right front wheel mass	34.2 (kg)
m_{w_2}	Left front wheel mass	34.2 (kg)
m_{w_3}	Right rear wheel mass	41.2 (kg)
m_{w_4}	Left rear wheel mass	41.2 (kg)
m_s	Mass of the seat	28 (kg)
k_{w_1}	Right front wheel stiffness	25.6 (kNm^{-1})
k_{w_2}	Left front wheel stiffness	25.6 (kNm^{-1})
k_{w_3}	Right rear wheel stiffness	17.3 (kNm^{-1})
k_{w_4}	Left rear wheel stiffness	17.3 (kNm^{-1})
c_{w_1}	Right front wheel damping	2.378 ($kNsm^{-1}$)
c_{w_2}	Left front wheel damping	2.378 ($kNsm^{-1}$)
c_{w_3}	Right rear wheel damping	1.604 ($kNsm^{-1}$)
c_{w_4}	Right rear wheel damping	1.604 ($kNsm^{-1}$)
k_{t_1}	Right front tyre stiffness	150 (kNm^{-1})
k_{t_2}	Left front tyre stiffness	150 (kNm^{-1})
k_{t_3}	Right rear tyre stiffness	150 (kNm^{-1})
k_{t_4}	Left rear tyre stiffness	150 (kNm^{-1})

c_{t_1}	Right front tyre damping	1.5 ($kNsm^{-1}$)
c_{t_2}	Left front tyre damping	1.5 ($kNsm^{-1}$)
c_{t_3}	Right rear tyre damping	1.5 ($kNsm^{-1}$)
c_{t_4}	Left rear tyre damping	1.5 ($kNsm^{-1}$)
L_1	Distance of center of gravity from front axle	1.4 (m)
L_2	Distance of center of gravity from rear axle	1.47 (m)
L_3	Distance of center of gravity from right axle	0.7 (m)
L_4	Distance of center of gravity from left axle	0.75(m)
